# Ultrasound-assisted extraction as an easy-to-perform analytical methodology for monitoring ibuprofen and its main metabolites in mussels

**DOI:** 10.1007/s00216-022-04153-w

**Published:** 2022-06-04

**Authors:** José Luis Malvar, Juan Luis Santos, Julia Martín, Irene Aparicio, Tainá Garcia Fonseca, Maria João Bebianno, Esteban Alonso

**Affiliations:** 1 Departamento de Química Analítica, Escuela Politécnica Superior, Universidad de Sevilla, C/Virgen de África, 7, E–41011 Seville, Spain; 2 CIMA, Centre for Marine and Environmental Research, University of Algarve, Campus de Gambelas, 8000-139 Faro, Portugal

**Keywords:** Ibuprofen, Metabolites, Mussels, Ultrasound-assisted extraction, Dispersive solid-phase extraction, Liquid chromatography-tandem mass spectrometry

## Abstract

Non-steroidal anti-inflammatory drugs (NSAIDs) have been reported to be the main pharmaceutical class accumulated in seafood. Among them, ibuprofen (IBU) is of special concern as it is used worldwide to treat common pain, does not require a medical prescription, it is often taken in a high daily dose, and has been reported to cause potential adverse effects on aquatic organisms. IBU is highly transformed into hydroxy- and carboxy-metabolites and/or degradation products generated not only after its administration but also during wastewater treatment or in the environment. These compounds can be present in the environment at higher concentrations than IBU and present higher toxicity. In this work, a low-cost and affordable routine analytical method was developed and validated for the first-time determination of IBU and its main metabolites in mussels. The method is based on ultrasound-assisted extraction (UAE), clean-up by dispersive solid-phase extraction (d-SPE) and analytical determination by liquid chromatography-tandem mass spectrometry. Box-Behnken experimental design was used for method optimisation to better evaluate the influence and interactions of UAE and d-SPE variables. Extraction recoveries were in the range from 81 to 115%. Precision, expressed as relative standard deviation, was lower than 7%. Method detection limits were in the range from 0.1 to 1.9 ng g^−1^ dry weight. The method was successfully applied to wild mussels.

**Graphical abstract:**

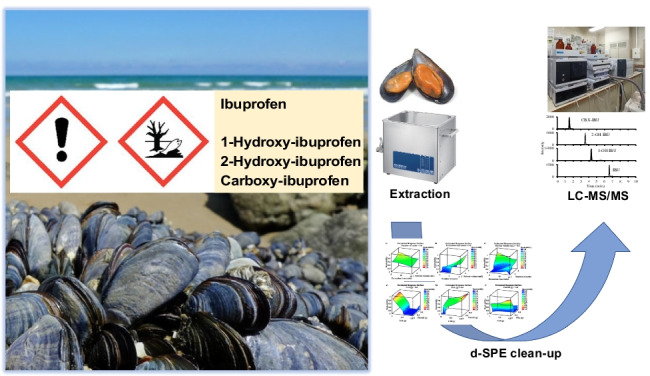

**Supplementary Information:**

The online version contains supplementary material available at 10.1007/s00216-022-04153-w.

## Introduction

Special attention has been paid in the last years to the presence of pharmaceutical compounds in the aquatic environment because of their biological activity, pseudo-persistence, bioaccumulation properties, and toxicity [1–3]. They are continuously discharged from wastewater treatment plants (WWTPs) [1, 4] and can be accumulated [2, 5–10] and trophic magnified [11] in aquatic organisms affecting not only the aquatic fauna [1, 12, 13] but also human health through the consumption of edible aquatic organisms [11, 14]. Their identification in marine bivalves is an issue of special interest as they are filter feeders that can accumulate pollutants present in the aquatic media [15–17] what makes them useful indicators of water pollution [18]. In addition, their accumulation in some marine bivalves, such as mussels, can result not only in antioxidant stress and endocrine disruption in mussels [6, 13, 19] but also constitute a way to entry into the human food chain as they are widely consumed seafood [20].

Nevertheless, to date, the determination of pharmaceutical compounds in mussels has mainly been limited to parent compounds [17, 21] in spite that their metabolites can be present in the aquatic environment at higher concentrations and may have higher toxicity [22, 23]. In addition, the scarce methods reported for the analysis of parent compounds and their metabolites in mussels are limited to antibiotics [24], carbamazepine (antiepileptic drug) [25, 26], diclofenac (anti-inflammatory drug) [27, 28], and fluoxetine (antidepressant drug) [3]. Such determinations are based on QuEChERS (quick, easy, cheap, effective, rugged, and safe) method [24–26, 28]; pressurised-liquid extraction (PLE) [27]; solid–liquid extraction [20]; and on ultrasound-assisted extraction (UAE) [3]. Extract clean-up, required due to the complexity of mussel matrix, it is usually carried out by solid-phase extraction (SPE) [3, 20, 27]. Analytical determination is commonly carried out by liquid chromatography-tandem mass spectrometry (LC–MS/MS) [24–26, 28] but gas chromatography-tandem mass spectrometry (GC–MS), after derivatisation [21, 27], and liquid chromatography with fluorimetric and diode array detectors have been also applied [6].

Among pharmaceutical compounds, non-steroidal anti-inflammatory drugs (NSAIDs) constitute a therapeutic group of special concern in the environment not only because of their ubiquitous presence in the aquatic media but also because they are the main pharmaceutical class accumulated in seafood [28]. Their common presence in the environment can be explained because they are administered at high doses [29] and can be affected by overuse [23] as they are sold without prescription and are used to treat common pain and inflammation what ease self-medication. Several studies have reported the presence of IBU and its metabolites in environmental waters, including wastewater effluents and seawater, at concentrations ranging from hundreds ng L^−1^ to thousands µg L^−1^ [1, 30–32] and it has been reported that IBU induces antioxidant stress and endocrine disruption in mussels [19]. Nevertheless, methods reported for the determination of NSAIDs and their metabolites in mussels are limited to diclofenac [27, 28] whereas methods reported for the determination of IBU in mussels are scarce [17, 21, 28]. In addition, no method has been reported yet for the determination of IBU metabolites in mussels in spite that they can be present at higher concentrations than IBU because just 15% of IBU is excreted unchanged [22]. Only a few papers report IBU concentrations in mussels [17, 21, 28, 33] and most of them report concentrations in the few samples used to test method applicability [21, 28]. Wolecki et al. reported IBU concentrations of 730 ± 290 (*n* = 5) in 2–3 cm mussels but it was not detected in < 3 cm individuals from the same sampling location. Mezzelani et al. [17] found IBU in 19% (*n* = 205) of mussels collected in 41 sampling campaigns from different locations along Italian coasts and at different seasons from 2014 to 2017. IBU concentration in contaminated samples was in the range from 9.4 to 143.7 ng g^−1^ d.w. IBU was detected in some sampling campaigns but not in others but no seasonal dependence was observed. The aim of this work was to develop and validate an affordable and easy-to-perform analytical method for the simultaneous determination of IBU and its main metabolites in mussels to cover the lack of analytical methods for such determination. This paper has been focused on IBU and its metabolites because, firstly, IBU is on the most used NSAIDs; secondly, it is administered at higher daily doses than others NSAIDs (IBU: 1.2 g day^−1^; naproxen: 0.5 g day^−1^; diclofenac: 0.1 g day^−1^) according to the World Health Organization ATC/DDD Index 2022 and has been widely used on COVID-19 patient treatment; thirdly, it has been reported to cause adverse effects on aquatic organisms within measured concentrations in surface waters [29, 34]; fourthly, it has been scarcely evaluated in aquatic organisms; and finally, no information about their metabolites in aquatic organisms has been reported yet. The proposed method is based on UAE, extract clean-up by dispersive solid-phase extraction (d-SPE) and analytical determination by LC–MS/MS. To the best of our knowledge, the proposed method is the first one to determine IBU and its metabolites in mussels.

## Materials and methods

### Chemicals and reagents

HPLC-grade methanol (MeOH), acetonitrile (ACN), acetone (ACE), and water were supplied by Romil (Barcelona, Spain). Analytical-grade formic acid (98%) was provided by Panreac (Barcelona, Spain). Analytical standards of IBU, 1-hydroxy-ibuprofen (1-OH-IBU), 2-hydroxy-ibuprofen (2-OH-IBU), carboxy-ibuprofen (CBX-IBU), and isotopically labelled ibuprofen-d_3_ (IBU-d_3_) were provided by Sigma-Aldrich (St. Louis, MO, USA). Physico-chemical properties of the target compounds are shown in Table 1. Individual stock solutions (1000 µg mL^−1^) and a mixture solution of the target compounds at 10 µg mL^−1^ each were prepared in MeOH and stored at − 18 °C. Working solutions at different concentration levels were prepared immediately before their use by dilution of the concentrated mixture solution (10 µg mL^−1^). Primary-secondary amine bonded silica (PSA) and octadecyl functionalised silica (C18) were provided by Scharlab (Barcelona, Spain). Florisil® was supplied by Sigma-Aldrich (St. Louis, MO, USA).

**Table 1:**
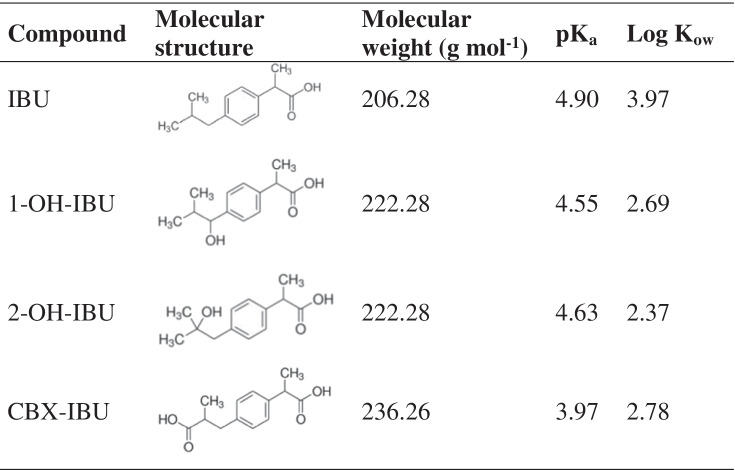
Molecular structure and main physical–chemical properties of the target compounds

### Sampling and sample pre-treatment

*Mytilus galloprovincialis* mussels used for method optimisation and validation were purchased from local markets. The proposed method was applied to wild *Mytilus galloprovincialis* mussels hand collected from Ria Formosa Lagoon, Southeast of Portugal. Mussels were transported alive to the laboratory. First, the shell was washed and then whole soft tissues were separated from the shell, frozen at − 18 °C, and lyophilised for 72 h in a Cryodos-50 lyophilizer (Telstar, Terrasa, Spain). Dried samples were crushed and homogenised in a mortar, sieved (particle size < 1 mm), and stored at − 18 °C in glass containers until analysis.

### UAE + d-SPE method

Lyophilised mussels (0.5 g d.w.) were transferred into glass centrifuge tubes and 5 mL of MeOH containing formic acid 0.5% v/v were added. The tubes were sonicated in an ultrasonic bath for 5 min and centrifuged at 2900 × *g* for 5 min. The liquid phase was transferred to a clean tube and the extraction procedure was repeated to complete three extraction cycles. Extracts were combined in the same tube for d-SPE clean-up by addition of Florisil® (0.8 g) and C18 (0.8 g) sorbents. The tubes were vortex-mixed for 1 min and centrifugated at 2900 × *g* for 10 min. The supernatants were transferred to clean tubes and evaporated to dryness at 50 °C under a gentle nitrogen stream in an XcelVap® automated evaporation/concentration system (Horizon Technology, Salem, New Hampshire, USA). Dried extracts were reconstituted by adding 250 µL of MeOH:water solution (1:1, v/v), filtered through a 0.22 µm cellulose syringe filter, and collected into an automatic injection vial for chromatographic analysis.

### LC–MS/MS determination

Chromatographic determination was carried out on an Agilent 1200 series HPLC (Agilent, USA) equipped with a vacuum degasser, a binary pump, an automatic injector, and a thermostatted column compartment. LC equipment was coupled to a 6410-triple quadrupole (MS/MS) mass spectrometer (MS) with an electrospray ionisation source (Agilent, USA). Chromatographic separation was carried out in a HALO C18 column (50 mm × 4.6 mm i.d., 2.7 µm particle size) (Advanced Materials Technology, USA) protected by a HALO C18 guard column (4.6 × 5 mm, 2.7 µm particle size) (Advanced Materials Technology, USA). Injection volume was 10 µL. The mobile phase was composed of ammonium acetate 10 mM (solvent A) and MeOH (solvent B). Gradient elution was carried out at 0.6 mL min^−1^ by linear increase of solvent B from 30 to 98% in 7 min. Back to initial conditions was produced by linear decrease of solvent B from 98 to 30% in 1 min, held for 2 min for re-equilibration. MS parameters were as follows: drying-gas flow rate, 9 L min^−1^; capillary voltage, 4000 V; drying-gas temperature, 350 °C; and nebuliser pressure, 40 psi. MS analysis was performed in multiple reaction monitoring (MRM) mode in both positive and negative ionisation modes. Table 2 shows MS parameters for each target compound. MassHunter software (Agilent, USA) was used for instrument control and data acquisition.

**Table 2:** LC–MS/MS parameters

Compound	Ionisation mode	Precursor ion (*m/z*)	Product ions (MRM1/MRM2) (*m/z*)	Fragmentor (V)	CE (eV)	RT (min)
IBU	Negative	205.1	161.1	64	0	6.50
1-OH-IBU	Positive	240.2	205.1/107.0	64	4/32	4.15
2-OH-IBU	Positive	240.2	205.1/107.0	64	8/36	3.32
CBX-IBU	Negative	235.1	191.1/73.1	64	0/8	1.08
IBU-d3	Negative	208.1	164.2	64	4	6.50

*CE*, collision energy; *RT*, retention time

## Results and discussion

### LC–MS/MS optimisation

LC–MS/MS optimisation was carried out by direct injection of 10 µg mL^−1^ individual standard solutions of the target compounds. The optimisation was carried out in both negative and positive modes using MeOH as organic solvent and ammonium acetate 10 mM or formic acid (0.1%, v/v) as aqueous solvents. IBU was better ionised when ammonium acetate was used whereas 1-OH-IBU and 2-OH-IBU were similarly ionised with both aqueous phases and CBX-IBU was just slightly better ionised when formic acid was used. From such results, ammonium acetate solution was selected as mobile phase aqueous solvent. IBU and CBX-IBU were better ionised in negative mode whereas 1-OH-IBU and 2-OH-IBU provided better results in positive ionisation mode. Two product ions were obtained for each compound except for IBU, as reported by other authors [28, 35]. The product ions with the highest intensities (MRM1) were used for quantification whereas the others (MRM2) were used for confirmation. In Figure S3, in Supplementary Information, it can be seen the chromatogram of a matrix-matched standard (100 µg L^−1^).

### Method optimisation

The most significant parameters affecting the extraction of the target compounds (extraction solvent, number of extraction cycles, extraction time, and solvent volume) and d-SPE clean-up (type and amount of dispersive sorbent) were evaluated. All the experiments were carried in triplicate with dry mussel tissues (0.5 g d.w.) spiked with the target compounds at a concentration of 125 ng g^−1^ d.w. each. Mussels were spiked by adding a standard solution in MeOH. After spike, they were left at room temperature during 24 h for MeOH evaporation. Results were evaluated by means of overall recoveries obtained by comparing signals obtained from spiked samples with signals from target compounds in pure solvent.

#### Extraction solvent optimisation

**Fig. 1:**
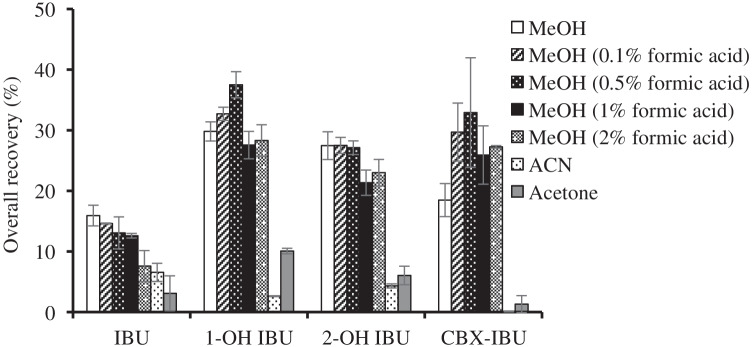
Influence of the type of extraction solvent on overall recoveries. Data obtained from mussels spiked at 125 ng g^−1^ d.w. in triplicate

First, the most critical extraction parameter, it is the type of extraction solvent, was optimised. A protic solvent (MeOH) and two aprotic solvents (ACN and ACE) were tested. Lyophilised samples (0.5 g) were weighed into centrifuge glass tubes and 4 mL of the evaluated extraction solvent were added to the tubes. The tubes were sonicated for 15 min and centrifuged at 2900 × *g* for 5 min. The liquid phases were transferred to clean tubes. Extracts were subjected to d-SPE clean-up by addition of 0.8 g of C18. The type and amount of clean-up sorbent were selected from previous studies reported for the determination of emerging pollutants in mussels based on d-SPE clean-up [36]. After C18 addition to the tubes, they were hand-shaken for 1 min and centrifuged at 2900 × g for 10 min. The extracts were transferred into clean centrifuge tubes and were evaporated to dryness by a gentle nitrogen stream. Dry extracts were dissolved in MeOH:water mixture (1:1, v/v), filtered through a 0.22 μm cellulose syringe filter, and collected in an automatic injection vial for LC–MS/MS analysis. As can be seen in Fig. 1, the best results for IBU, 1-OH-IBU, and 2-OH-IBU were obtained when MeOH was used whereas CBX-IBU was better extracted with ACN. Overall recoveries lower than 15% were obtained when ACE was used. As can be seen in Table 1, IBU and its metabolites are acid compounds with one or two carboxylic groups in their structure and pKa values in the range from 3.97 to 4.90. Therefore, the influence of the acidification of MeOH with formic acid at different concentration levels (0.1%, 0.5%, 1%, and 2%, v/v) was tested. The acidic conditions were expected to promote the unionised form of the target compounds easing their transference to the organic extraction solvent. The overall recoveries of the most acidic compounds (CBX-IBU and 1-OH-IBU) increased when formic acid content was increased from 0 to 0.5%, v/v (Fig. 1). At higher concentrations of formic acid, the overall recoveries decreased. This fact could be explained by the extraction of interfering compounds causing signal suppression (matrix effect). Overall recoveries of IBU decreased slightly when formic acid was added to MeOH. Therefore, MeOH acidified with formic acid (0.5%, v/v) was selected as extraction solvent.

#### Extraction time, cycles, and solvent volume optimisation

Once the type of extraction solvent was selected, the other parameters involved in sample extraction (sonication time, extraction solvent volume, and number of extraction cycles) were optimised by a Box-Behnken design (BBD). The experimental design allows not only to reduce the number of experiments but also to better evaluate the influence of each variable and their interactions in overall recoveries. The number of experiments was determined by applying Eq. (1):

1 $$N=2k\left(k-1\right)+{C}_{0}$$

where *N* is the number of experiments, *k* is the number of variables, and C_0_ is the number of central points. In this experiment, the number of variables (*k*) to optimise was 3 (number of cycles, extraction time, and solvent volume) and one central point was incorporated in triplicate (C_0_ = 3). Each variable was evaluated at three levels (number of cycles: 1, 2, and 3; extraction time: 5, 10, and 15 min; solvent volume: 3, 4, and 5 mL). The number of experiments required was 15. They were randomly performed to reduce the effects of uncontrolled variables. In Table S1 in Supplementary Information, it can be seen values for each variable in each experiment. The influence of the variables in overall recovery of each target compound was evaluated using ANOVA test and by standardised (*P* = 0.05) Pareto charts. Pareto charts (Figure S1 in Supplementary Information) demonstrated that the number of extraction cycles was the most significant variable affecting overall recoveries, with positive effect, mainly in the case of CBX-IBU. No remarkable effects related to extraction time and solvent volume were observed for any of the selected compounds.

**Fig. 2:**
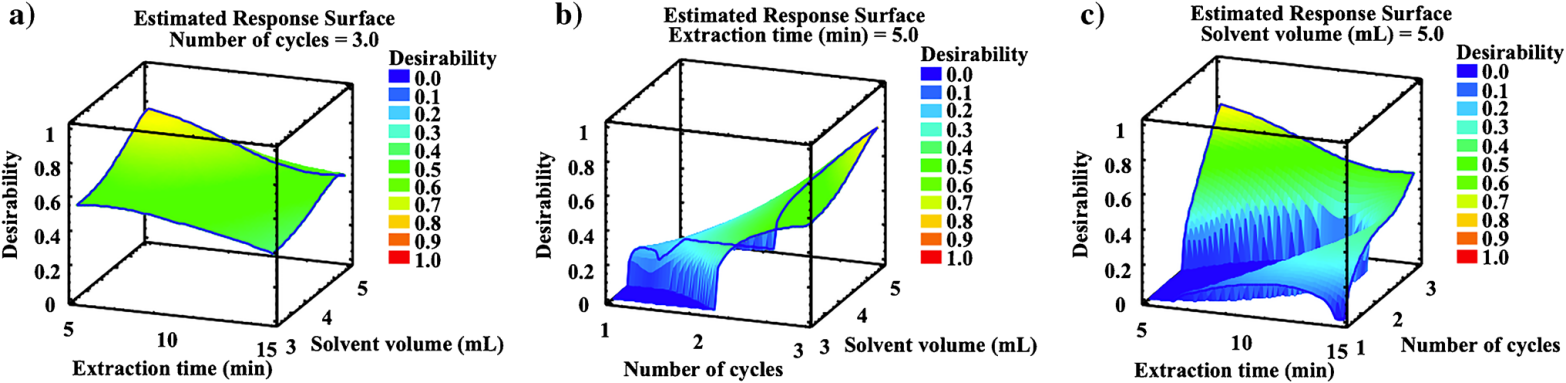
Response surface plots corresponding to the global desirability function for UAE optimisation. Data obtained from mussels spiked at 125 ng g^−1^ d.w. in triplicate

Estimated response surface, corresponding to the global desirability function, was plotted to evaluate the effects of the variables and their interactions on overall recoveries. The global desirability functions were defined as the geometric mean value of the normalised responses, it is the overall recovery values of the four target compounds. Statgraphics Plus software version 5.1 (Statpoint Technologies Inc., Warrenton, VA, USA) was used for statistical treatment of the data. In Fig. 2, estimated response surface plots corresponding to the global desirability function versus extraction time and solvent volume (Fig. 2a); number of extraction cycles and solvent volume (Fig. 2b); and extraction time and extraction cycles (Fig. 2c) are shown. As can be seen in these figures, the highest values of the global desirability function were obtained for 3 extraction cycles, 5 min of extraction time, and 5 mL of extraction solvent. Therefore, such values were selected as the best extraction conditions.

#### Optimisation of d-SPE clean-up

**Fig. 3:**
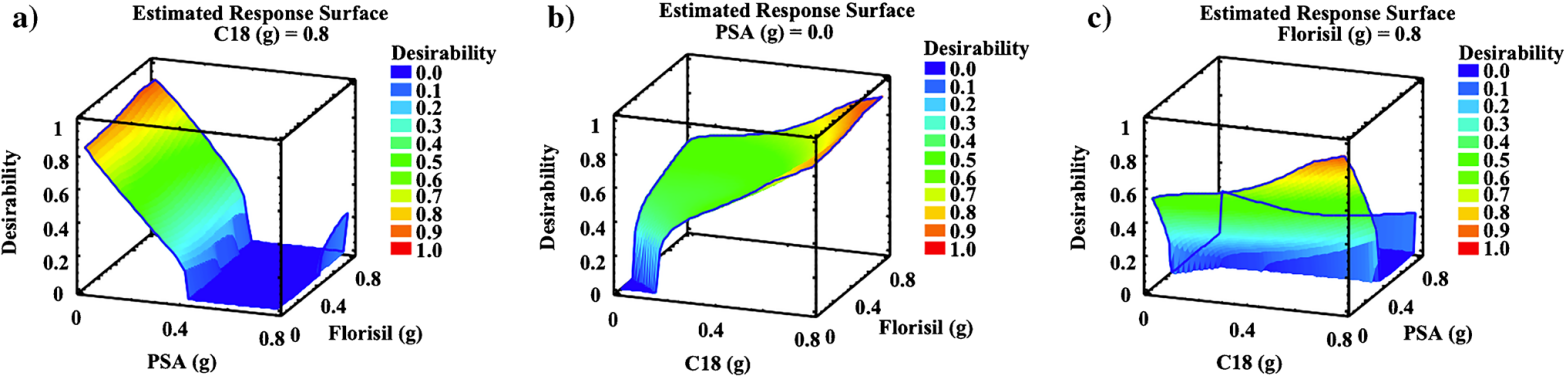
Response surface plots corresponding to the global desirability function for d-SPE clean-up optimisation. Data obtained from mussels spiked at 125 ng g^−1^ d.w. in triplicate

A reverse-phase sorbent (C18), a mixed-mode sorbent (PSA), and a normal-phase sorbent (Florisil®) were evaluated as clean-up sorbents. The tested sorbents were selected because of their capacity to remove nonpolar compounds (C18), nonpolar to moderately polar compounds (PSA), and polar compounds (Florisil®). BBD design was applied to select the best clean-up sorbents and their amounts. The variables (type of sorbent: C18, PSA, and Florisil®) were evaluated at three levels (sorbent amount: 0, 0.4, and 0.8 g). The levels were selected according to the good results previously obtained in the determination of IBU and its metabolites in other complex solid matrices [37]. In Table S2 in Supplementary Information, it can be seen values for each variable in each experiment. Pareto charts revealed that PSA sorbent was the most significant variable presenting a strong negative effect on the overall recoveries of CBX-IBU whereas Florisil® presented positive effects for 1-OH IBU and 2-OH IBU and C18 showed no remarkable effects for none of the target compounds (Figure S2, in Supplementary Information). In Fig. 3, estimated response surface plots corresponding to the global desirability function versus PSA and Florisil® (Fig. 3a); C18 and Florisil® (Fig. 3b); and C18 and PSA (Fig. 3c) are shown. As can be seen in Fig. 3, the highest values of the global desirability function were achieved when a high amount of C18 and Florisil® and a low amount of PSA were used. Therefore, 0.8 g of C18, 0.8 g of Florisil®, and 0 g of PSA were selected as the best sorbents and amounts for d-SPE extract clean-up.

### Method validation

The optimised method was validated in terms of linearity, selectivity, recovery, precision, method detection limit (MDL), and method quantification limit (MQL). Matrix effect was qualitatively evaluated by comparing slopes of matrix-matched calibration curves, obtained from spiked mussel extracts, with external calibration curves, obtained from standard solutions in pure solvent. In both cases, eleven-point calibration curves were prepared in the range from 0.5 to 500 µg L^−1^ using MeOH:water (1:1, v/v) as solvent. The differences between calibration curve slopes were statistically evaluated by applying Student’s *t*-test at 95% of confidence. The test revealed significant differences between slopes caused by matrix effect. Therefore, matrix-matched calibration curves must be used for quantitation. Matrix effect (ME, %) was quantified at four concentration levels (25, 50, 125, and 250 ng g^−1^ d.w.) by comparing the area of the target compounds in mussel extract (*A*_extract_), after subtracting non-spiked extract signal (*A*_blank_), and in MeOH:water (1:1, v/v) (*A*_standard_) following Eq. (2) :

2 $$ME\left(\mathrm{\%}\right)\;=\left({A}_{extract}-{A}_{blank}\right)/{A}_{standard}\times 100$$

Average matrix effects for each target compound are shown in Table 3. All the compounds were affected by strong signal suppression, with matrix effect in the range from − 50 to − 82%. The use of an isotopically labelled compound (IBU-d_3_) was tested as internal standard to compensate matrix effect. Matrix effect was reduced for IBU but not for its metabolites. Matrix effect affecting IBU metabolites was reduced just from 10 (for CBX-IBU) to 30% (for 1-OH-IBU) by the use of the internal standard. Therefore, the correction of matrix effect had to be carried out by matrix-matched calibration curves as reported by other authors [33, 35, 36]. Nevertheless, the internal standard was used to compensate residual potential residual matrix effects [36].

**Table 3:** Matrix effect (ME), instrumental detection limits (IDLs), instrumental quantification limits (IQLs), linear dynamic ranges (LDRs), correlation coefficients (*R*^2^), method detection limits (MDLs), and method quantification limits (MQLs)

Compound	ME (%)	IDL (ng mL^−1^)	IQL (ng mL^−1^)	LDR (ng mL^−1^)	*R*^2^	MDL (ng g^−1^ d.w.)	MQL (ng g^−1^ d.w.)
IBU	− 82	1.5	5	5–500	0.998	0.7	2.4
1-OH-IBU	− 56	0.3	1	1–500	0.999	0.1	0.5
2-OH-IBU	− 50	1.5	5	5–500	0.999	0.7	2.4
CBX-IBU	− 70	3.0	10	10–500	0.999	1.9	6.2

Instrumental detection limits (IDLs) and instrumental quantification limits (IQLs) were estimated by spiking mussel extracts at low concentration levels. They were fixed at the lowest concentrations corresponding to signal-to-noise ratios of 3 and 10, respectively. MDLs and MQLs were calculated from IDLs and IQLs, respectively, by taking into account concentration factor achieved by sample treatment and extraction recovery of each compound. MDL and MQL values were in the range from 0.1 to 1.9 ng g^−1^ d.w. and from 0.5 to 6.2 ng g^−1^ d.w., respectively (Table 3). Recovery and precision were evaluated from spiked mussels at four concentration levels (25, 50, 125, and 250 ng g^−1^ d.w.) in triplicate. Recovery was estimated by comparing the areas of the target compounds in spiked extracts with those obtained from spiked samples. The precision, expressed as relative standard deviation (RSD, %), was determined in terms of reproducibility by measuring the spiked samples in triplicate in three different days. Recoveries were in the range from 81 to 115% in all cases whereas precision, expressed as RSD (%), was below 7% for all the target compounds and concentration levels (Table 4). In Table 5, it can be seen a comparison between the proposed method and the scarce methods reported for the determination of IBU in bivalves. Mezzelani et al. [17] proposed a method based on HPLC–DAD/Fl determination that provided recoveries and MDLs similar to those of the proposed method. Nevertheless, it required higher sample amounts (3 g) and longer sample treatment times (70 min). The proposed method provides recovery and MDL values similar to those reported by PLE [21] but such extraction method requires the use of a high-cost equipment and longer sample treatment (94 min). QuEChERS method has been proposed by Mello et al. [28] and Núñez et al. [33] for the determination of IBU and other pharmaceutical active compounds in bivalves. The method reported by Mello et al. [28] provided similar recoveries and threefold lower MDL for IBU but poorer linearity, just in the range from 3.5 to 35.75 ng g^−1^ d.w., and higher ME (+ 176% for IBU) whereas the method reported by Núñez et al. [33] provided MDL and MQL of IBU 71-fold higher than those of proposed method. No comparison can be done for metabolites of IBU because, to the best of our knowledge, no method has been reported yet for their determination in mussels.

**Table 4:** Recovery (R) and precision, expressed as relative standard deviation (%, RSD), at four spike concentration levels

Compound	25 ng g^−1^ d.w		50 ng g^−1^ d.w		125 ng g^−1^ d.w		250 ng g^−1^ d.w	
	R	RSD	R	RSD	R	RSD	R	RSD
	(%)	(%)	(%)	(%)	(%)	(%)	(%)	(%)
IBU	103	6	115	2	107	7	110	3
1-OH-IBU	104	7	92	5	98	2	101	3
2-OH-IBU	105	1	88	3	97	4	94	2
CBX-IBU	81	2	104	2	93	6	84	7

**Table 5:** Analytical methods reported for the determination of IBU in bivalves

Compounds	Sample amount (g)	Extraction technique	Sample treatment time (min)	Clean-up	Analytical determination	R^a^ (%)	MDL^a^ (ng g^−1^ d.w.)	Reference
IBU (Others: 3 NSAIDs, 1 analgesic, 3 psychiatric drugs, 1 antihypertensive)	3	Solid–liquid extraction in a dispersing, stirring, homogenising, and grinding system	70	SPE	HPLC–DAD/FL	95–96	8^b^	[17]
IBU (Others: 4 NSAIDs and 3 estrogenic hormones)	0.1	PLE	94	SPE	GC–MS (after derivatisation)	82–105	1	[21]
IBU (Others: 3 NSAIDs, 1 antibiotic, 1 anticonvulsant, 2 diuretic, and 3 antilipidemic agents; 1 metabolite of DIC)	0.5	QuEChERS	No data	-	LC–MS/MS	88–104	0.22	[28]
IBU (Others: 3 NSAIDs; 1 lipid regulator; the metabolite of an analgesic and the metabolite of a lipid regulator)	1	QuEChERS	13	-	LC–MS/MS	90	50	[33]
IBU and 3 of its metabolites	0.5	UAE	40	d-SPE	LC–MS/MS	103	0.7	Proposed method

^a^R and MDL values correspond to IBU; ^b^Corresponds to method quantitation limit

*d-SPE*, dispersive solid-phase extraction; *HPLC–DAD/FL*, high performance liquid chromatography with diode array and fluorimetric detection; *IBU*, ibuprofen; *MDL*, method detection limit; *NSAID*, non-steroidal anti-inflammatory drug; *PLE*, pressurised liquid extraction; *R*, recovery; *SPE*, solid-phase extraction; *UAE*, ultrasound-assisted extraction

### Method application

The validated method was applied to wild *Mytilus galloprovincialis* mussels (*n* = 10) hand collected from Ria Formosa Lagoon, Southeast of Portugal. None of the target compounds was detected in the analysed mussels. Same results were reported for mussels collected from the Atlantic Coast of France, Spanish SE Mediterranean Coast, and Spanish NE Atlantic Coast and purchased from a local market [35]. Nevertheless, high concentrations of IBU have been reported in mussels collected from the Gulf of Gdansk (southern Baltic Sea) (730 ± 290 ng g^−1^ d.w. in 2–3 cm mussels and lower than MDL in > 3 cm mussels) (*n* = 5) [21] and Adriatic Sea (from lower than 8 ng g^−1^ d.w. (MQL), in 80% of the 205 analysed mussels, to up to 143.7 ± 242.0 ng g^−1^ d.w.) [17]. Among the five non-steroidal anti-inflammatory drugs determined in mussels by Wolecki et al. [21], IBU was the one at the highest concentrations in 2–3 cm individuals (IBU: 730 ± 290 ng g^−1^ d.w.; naproxen: 473 ± 76 ng g^−1^ d.w.; diclofenac: 560 ± 130 ng g^−1^ d.w.; paracetamol and ketoprofen: non-detected) whereas none of the compounds was detected in > 3 cm individuals. Lower concentrations (mean values: 1.78 ng g^−1^ d.w.) were reported for IBU in mussels from Sepetiba Bay (Southeastern coast of Brazil) [28]. Nevertheless, among the eleven pharmaceutical compounds determined in five types of seafood samples, IBU was the one most frequently detected (67% of the 149 analysed samples) with no significative differences among species and the one with the highest mean concentrations. No information has been reported yet about the presence of IBU metabolites in mussels. They could be present at higher concentrations than IBU in those mussels where IBU was detected as 85% of IBU is excreted as metabolites [22].

## Conclusions

An analytical method has been developed and validated for the first-time determination of IBU and its main metabolites in mussels. The method is based on UAE followed by d-SPE clean-up and LC–MS/MS determination. Sample treatment proposed is easy-to-perform and do not require sophisticated instrumentation what makes it suitable to be applied for monitoring IBU parent compound and metabolites in mussels. Recoveries were in the range from 81 to 115% for all compounds. Precision, expressed as relative deviation standard (%, RSD), was below 7% for all the target compounds and spike levels. MDLs values were in the range from 0.1 to 1.9 ng g^−1^ d.w. The proposed method can constitute a useful tool be used (i) to obtain information about water pollution by using mussels as bioindicators; (ii) to evaluate the effects of such compounds in mussels by means of controlled exposure experiments; and (iii) for food safety controls.

## Supplementary Information

Below is the link to the electronic supplementary material.

Supplementary file1 (DOCX 993 KB)
