# Supplementary material for: Ultrasound-assisted extraction as an easy-to-perform analytical methodology for monitoring ibuprofen and its main metabolites in mussels
Source: Anal Bioanal Chem. 2022 Jun 4;414(19):5877–86. doi: 10.1007/s00216-022-04153-w (PMC9166212; doi:10.1007/s00216-022-04153-w)
Supplement: Supplementary file 1 — Supplementary file1 (DOCX 993 KB) [file 216_2022_4153_MOESM1_ESM.docx]

**Ultrasound-assisted extraction as an easy-to-perform analytical methodology for monitoring ibuprofen and its main metabolites in mussels**

José Luis Malvar^1^, Juan Luis Santos^1^, Julia Martín^1^, Irene Aparicio^1^, Tainá Garcia Fonseca^2^, Maria João Bebianno^2^, Esteban Alonso^1,*^

^1^Departamento de Química Analítica, Escuela Politécnica Superior, Universidad de Sevilla. C/ Virgen de África, 7, E–41011 Seville, Spain.

^2^CIMA, Centre for Marine and Environmental Research, University of Algarve, Campus de Gambelas, 8000-139, Faro, Portugal*.*

**Corresponding author**: Esteban Alonso

*E-mail:* ealonso@us.es

**Table S1.** Box–Behnken design matrix for optimization of number of extraction cycles, extraction time and solvent volume.

| Experiment | Extraction cycles  (n) | Extraction time  (min) | Solvent volume  (mL) |
| --- | --- | --- | --- |
| 1 | 3 | 10 | 5 |
| 2 | 3 | 10 | 3 |
| 3 | 2 | 5 | 5 |
| 4 | 1 | 10 | 5 |
| 5 | 2 | 15 | 5 |
| 6 | 2 | 10 | 4 |
| 7 | 1 | 15 | 4 |
| 8 | 3 | 15 | 4 |
| 9 | 1 | 5 | 4 |
| 10 | 2 | 5 | 3 |
| 11 | 2 | 10 | 4 |
| 12 | 2 | 10 | 4 |
| 13 | 3 | 5 | 4 |
| 14 | 2 | 15 | 3 |
| 15 | 1 | 10 | 3 |

**Table S2.** Box–Behnken design matrix for optimization of type and amount of d-SPE sorbent.

| Experiment | C18 amount  (g) | PSA amount  (g) | Florisil amount  (g) |
| --- | --- | --- | --- |
| 1 | 0.4 | 0 | 0.8 |
| 2 | 0.4 | 0.4 | 0.4 |
| 3 | 0.4 | 0.8 | 0.8 |
| 4 | 0.8 | 0.8 | 0.4 |
| 5 | 0.4 | 0.4 | 0.4 |
| 6 | 0.4 | 0 | 0 |
| 7 | 0.4 | 0.8 | 0 |
| 8 | 0.8 | 0.4 | 0.8 |
| 9 | 0 | 0 | 0.4 |
| 10 | 0.8 | 0.4 | 0 |
| 11 | 0.8 | 0 | 0.4 |
| 12 | 0 | 0.4 | 0.8 |
| 13 | 0 | 0.4 | 0 |
| 14 | 0 | 0.8 | 0.4 |
| 15 | 0.4 | 0.4 | 0.4 |

**Figure S1.** Standardized Pareto charts to evaluate the influence of UAE variables.

**Standardized Pareto Chart for IBU**

**0**

**1**

**2**

**3**

**4**

**5**

**Standardized effect**

**AC**

**C: Solvent volume**

**BC**

**A: Extraction time**

**B: Extraction cycles**

**AA**

**CC**

**BB**

**AB**

**+**

**-**

**Standardized Pareto Chart for 1-OH-IBU**

**0**

**0.5**

**1**

**1.5**

**2**

**2.5**

**3**

**Standardized effect**

**AC**

**A: Extraction time**

**AB**

**C: Solvent volume**

**BB**

**AA**

**CC**

**BC**

**B: Extraction cycles**

**+**

**-**

**Standardized Pareto Chart for 2-OH-IBU**

**0**

**0.5**

**1**

**1.5**

**2**

**2.5**

**3**

**Standardized effect**

**AC**

**A: Extraction time**

**AB**

**BB**

**C: Solvent volume**

**B: Extraction cycles**

**CC**

**AA**

**BC**

**+**

**-**

**Standardized Pareto Chart for CBX-IBU**

**0**

**1**

**2**

**3**

**4**

**5**

**Standardized effect**

**A: Extraction time**

**C: Solvent volume**

**AB**

**AC**

**BB**

**AA**

**CC**

**BC**

**B: Extraction cycles**

**+**

**-**

**Figure S2.** Standardized Pareto charts to evaluate the influence of d-SPE sorbents.

**Standardized Pareto Chart for IBU**

**0**

**0.5**

**1**

**1.5**

**2**

**2.5**

**3**

**Standardized effect**

**C: Florisil**

**AC**

**CC**

**B: PSA**

**AA**

**BC**

**BB**

**AB**

**A: C18**

**+**

**-**

**Standardized Pareto Chart for 1-OH-IBU**

**0**

**1**

**2**

**3**

**4**

**5**

**6**

**Standardized effect**

**AB**

**AC**

**A: C18**

**BC**

**B: PSA**

**CC**

**C: Florisil**

**AA**

**BB**

**+**

**-**

**Standardized Pareto Chart for 2-OH-IBU**

**0**

**3**

**6**

**9**

**12**

**15**

**Standardized effect**

**A: C18**

**AC**

**B: PSA**

**BC**

**C: Florisil**

**AB**

**BB**

**CC**

**AA**

**+**

**-**

**Standardized Pareto Chart for CBX-IBU**

**0**

**10**

**20**

**30**

**40**

**Standardized effect**

**AC**

**AA**

**CC**

**A: C18**

**AB**

**C: Florisil**

**BC**

**BB**

**B: PSA**

**+**

**-**

**Figure S3.** LC-MS/MS chromatogram of a 100 ng mL^-1^ matrix-matched calibration standard.
